# ARCHITECT HIV Combo Ag/Ab and RealTime HIV-1 Assays Detect Diverse HIV Strains in Clinical Specimens

**DOI:** 10.1089/aid.2017.0244

**Published:** 2018-03-01

**Authors:** Mary A. Rodgers, Ana S. Vallari, Julie Yamaguchi, Vera Holzmayer, Barbara Harris, Coumba Toure-Kane, Souleymane Mboup, Samar Badreddine, Carole McArthur, Nicaise Ndembi, Dora Mbanya, Lazare Kaptue, Gavin Cloherty

**Affiliations:** ^1^ Infectious Disease Research, Abbott Diagnostics, Abbott Park, Illinois.; ^2^ Institut de Recherche en Santé, de Surveillance Epidemiologique et de Formations, Dakar, Senegal.; ^3^ Infection Control Unit, King Faisal Specialist Hospital and Research Center, Jeddah, Saudi Arabia.; ^4^ Department of Oral and Craniofacial Sciences, School of Dentistry, University of Missouri-Kansas City, Kansas City, Missouri.; ^5^ Pathology Department, Truman Medical Center, Kansas City, Missouri.; ^6^ Laboratory Research Department, Institute of Human Virology, Abuja, Nigeria.; ^7^ Department of Hematology, Université de Yaoundé I, Yaoundé, Cameroon.; ^8^ Laboratoire D'Hematologie, Université des Montagnes, Bangangté, Cameroon.

**Keywords:** HIV diversity, diagnostics, serology, nucleic acid test

## Abstract

Periodic evaluation of the impact of viral diversity on diagnostic tests is critical to ensure current technologies are keeping pace with viral evolution. To determine whether HIV diversity impacts the ARCHITECT HIV Combo Ag/Ab (HIV Combo) or RealTime HIV-1 (RT) assays, a set of *N* = 199 HIV clinical specimens from Cameroon, Senegal, Saudi Arabia, and Thailand were sequenced and tested in both assays. The panel included historical groups N and P specimens and a newly identified group N specimen. These and specimens classified as H, U (unclassified)/URF (unique recombinant form), CRF (circulating recombinant form) 01, 02, 06, 09, 11, 13, 18, 22, 37, and 43 were detected by both the RT assay (1.75–6.84 log copies/ml) and the HIV Combo assay (3.26–1121.96 sample to cutoff ratios). Sequence alignment identified 3 or fewer mismatches to the RT assay oligos in 82.4% of samples. Altogether, these data demonstrate the HIV Combo and RT assays detect diverse strains of HIV in clinical specimens.

The deep genetic diversity of HIV-1 is primarily driven by an error-prone viral reverse transcriptase, high rates of recombination, and multiple cross-species transmission events.^1^ To date, 4 groups of HIV-1 (M, N, O, and P) have been identified, as well as 9 major subtypes (A, B, C, D, F, G, H, J, and K), and at least 90 circulating recombinant forms (CRFs) of group M (Los Alamos National Laboratories HIV database, date of accession: September 8, 2017). Local pandemics in some parts of the world are limited to 1 or 2 major strains of HIV, while regions of sub-Saharan Africa have reported all major strains in circulation.^1^ However, with increases in global travel, immigration, and military deployment, regions outside of sub-Saharan Africa are experiencing increases in HIV-1 strain diversity. Sequence comparisons indicate that the average nucleotide divergence between HIV-1 groups is 37.5%, with 14.7% between subtypes, and 8.2% within subtypes.^2^ This extensive sequence heterogeneity is a challenge for the design of therapeutics, vaccines, blood screening, and diagnostic tests, which rely fundamentally on sequence conservation.

The detection of diverse strains by the RealTime HIV-1 assay (RT; Abbott Molecular Diagnostics, Des Plaines, IL) and the HIV Combo Ag/Ab assay (HIV Combo; Abbott Diagnostics, Abbott Park, IL) reported in several recent publications largely relied on cultured virus isolates.^3–9^ In contrast to cultured virus isolates, clinical specimens are complex sample types that are representative of the samples tested in clinical diagnostic laboratories. Therefore, evaluation of the detection of diverse HIV-1 strains in collections of unique clinical specimens is an important challenge to determine whether sequence and specimen diversity may affect assay performance. For both the HIV Combo and RT assays, antigen-only cultured virus isolates have been used to demonstrate the detection of rare CRF, groups N, O, and P specimens,^4,5^ and clinical specimens have been tested for up to 13 different CRFs, group O, group P, and rare subtypes H, J, and K.^3,6–8,10^ However, clinical specimens for newly identified group N, group P, and CRF specimens must also be tested as these branches of the HIV phylogenetic tree continue to diversify. Toward this end, we have challenged the HIV Combo and RT assays with a diverse set of previously untested clinical HIV-1 specimens, including rare CRFs, group N, and group P specimens.

Specimens (*N* = 199) were collected by the Abbott Global Surveillance Program through collaborations in Cameroon, Saudi Arabia, Senegal, and Thailand (Fig. 1). All specimens were obtained according to local regulations in each country at the time of collection between 1998 and 2016, including local IRB approval when required. Specimens were identified as HIV-1 positive by rapid diagnostic tests done in source countries before sequence analysis and testing at Abbott Laboratories. Age and gender demographic information were available for the majority of participants, of which 67% were male and the median age was 34 years with an interquartile range of 23 (Fig. 1).

**FIG. 1.**
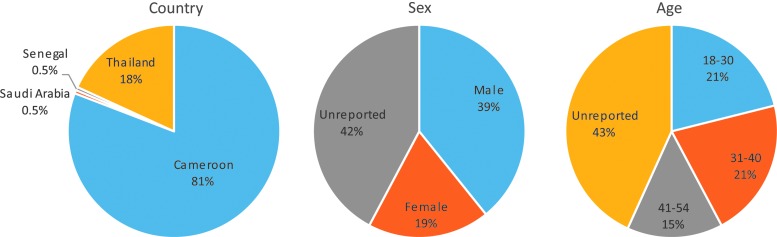
Summaries of demographics. The country, sex, and age at the time of specimen collection are plotted in pie charts as percentages of the total number of samples (*N* = 199). The full age range of the study cohort is 18–54 years with a median age of 34 years.

Specimens were sequenced as previously described in the polymerase integrase (*pol* IN) region and when specimen volume allowed, the *env* IDR region was also sequenced.^8^ Complete genome or subgenomic sequences for 14 rare specimens included in this study have been previously reported: group P specimen U14788,^11^ group N specimens S4858,^7^ U14296,^12^ and DJO0135,^13^ subtype H specimen K562,^7^ subtype G specimen J11254,^14^ and eight CRF specimens.^7^ Viral load data had not been collected for any of these samples when they were originally sequenced and only S4858, K562, and the CRF specimens have been previously tested by HIV Combo.^7^ The *pol* IN and *env* IDR sequences from these samples were included in the subsequent phylogenetic analysis to confirm subgenomic classifications with an updated reference sequence alignment before testing by the HIV Combo and RT assays.

The *pol* IN and *env* IDR sequences were classified by phylogenetic inference with reference sequences obtained from the Los Alamos National Laboratories database for SIVcpz, SIVgor, group N, group P, group O, group M subtypes A–K, and CRFs 1–90 as previously described.^7^ After removing the CRF references that did not branch with or near the specimen sequences, a final phylogenetic tree was built for each region sequenced as shown in Figure 2, and the classifications are summarized in Table 1. For specimens with different classifications in the two sequenced regions, the final classification of URF (unique recombinant form) was assigned. Notably, specimen J11254 was reclassified as CRF43 as it clearly branched with CRF43 reference sequences and one new group N specimen was identified, U22816, which is the 17th sequence confirmed group N infection reported to date (Fig. 3). U22816 was collected in 2012 from a 48-year-old female in Yaoundé, Cameroon, and it is basal to other group N sequences in both the *pol* IN and *env* IDR regions (Fig. 2).

**FIG. 2.**
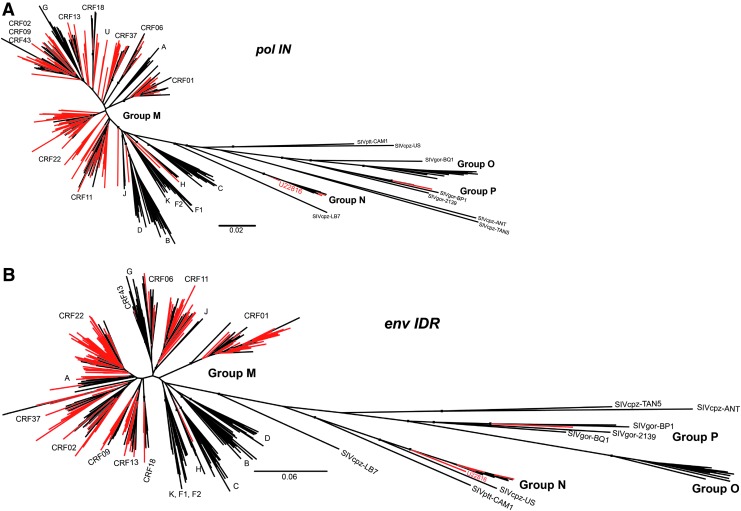
Phylogenetic trees. The phylogenetic trees for the *pol* IN **(**1009nt, **A)** and *env* IDR (676nt, **B**) are shown with specimen sequences indicated in *red* and reference sequences indicated in *black*. Relevant nodes with bootstrap values >70 are labeled by small *black boxes*. CRF, circulating recombinant form; U, unclassified.

**FIG. 3.**
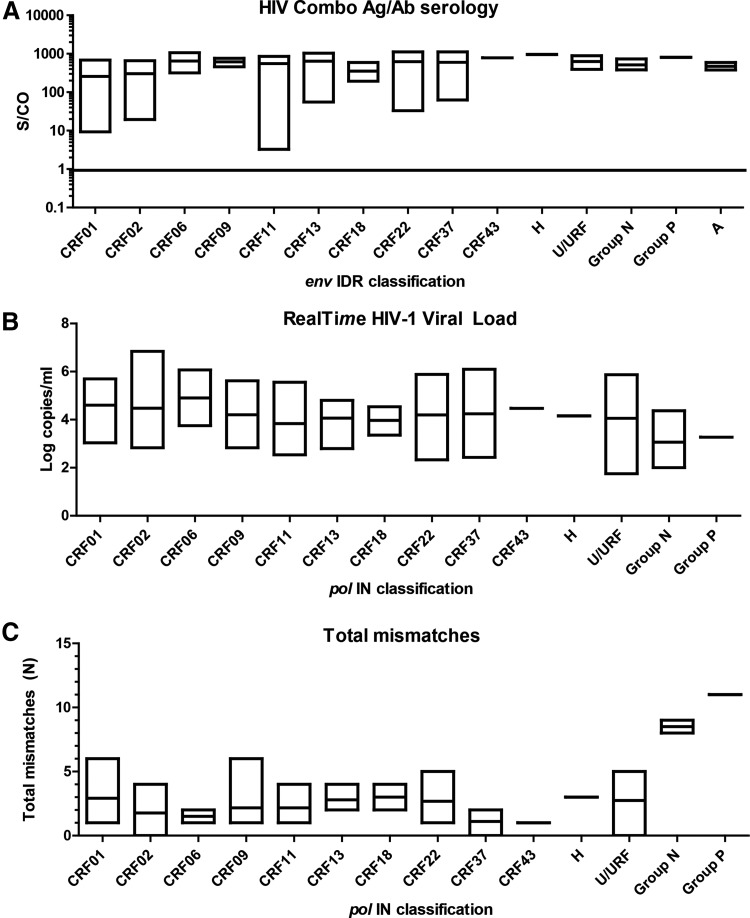
Detection of HIV infection in diverse clinical specimens. The range and mean viral load **(A)** and S/CO **(B)** detected from clinical specimens of each HIV classification are plotted. The classifications shown in **(A)** are for the *pol* IN region and the classifications indicated in **(B)** are for the *env* IDR region. The range and mean number of total mismatches in the HIV RealTime primers and probe are plotted for each *pol* IN classification in **(C)**. Only one clinical specimen was available for CRF43, H, and P. URF, unique recombinant form.

**Table 1.** Summary of Classifications

*Classification*	pol *IN*	env *IDR*	*Overall*
CRF01	36	36	36
CRF02	26	11	11
CRF06	4	9	4
CRF09	6	6	6
CRF11	24	25	18
CRF13	5	14	4
CRF18	3	3	3
CRF22	56	57	51
CRF37	9	15	8
CRF43	1	1	1
U/URF	23	7	51
H	1	1	1
A	0	4	0
Not sequenced	NA	5	NA
Group N	4	4	4
Group P	1	1	1
Total	199	199	199

The classifications determined by phylogenetic inference as shown for the *pol* IN and *env* IDR sequences. Overall classifications were determined by comparing *pol* IN and *env* IDR individual classifications. Where regions did not match, an overall classification of URF was made. *N* = 5 specimens could not be sequenced in the *env* IDR region. The overall classifications for these samples were determined solely by the *pol* IN sequence.

CRF, circulating recombinant form; NA, not applicable; U, unclassified; URF, unique recombinant form.

Since the HIV Combo assay targets a region of the *env* IDR and the RT assay targets a region of the *pol* IN, the classifications of these individual regions were used for challenging each respective assay. Notably, many specimens had sequences that did not branch with any references or were very basal to reference sequences (Fig. 2). These sequences were called unclassified (U) and recombinant analysis using Simplot software identified unique breakpoints, indicating they might be URFs.

All 199 samples were tested by the RT assay according to the manufacturer's protocol. The majority of specimens were tested neat, although specimens with limited volume were diluted 1:10 in HIV-negative human plasma before testing. The reported viral load (log copies/ml) values were adjusted to account for dilution factors. The log copies/ml result ranges and averages for each HIV *pol* IN classification are shown in Figure 3. All specimens were detected regardless of classification with a viral load ranging from 1.75 to 6.84 log_10_ copies/ml (Fig. 3). When the *pol* IN sequences were compared to the forward primer, probe, and reverse primer in the RT assay, the number of combined mismatches for all three regions ranged from 0 to 11 (Fig. 3), with the most mismatches in the group P sequence. The majority of specimens (*N* = 164, 82.4%) had three total mismatches or less, indicating that the region for the RT amplicon is well conserved. Importantly, all the samples were detected despite the identified primer and probe mismatches, indicating that the RT assay can accommodate the full breadth of existing HIV diversity.

Specimens were also tested in the HIV Combo assay. While most were tested neat, the rare groups N and P samples were diluted 1:10–1:50 and S/CO results were not adjusted for dilution since the assay is not quantitative. All specimens were reactive in the HIV Combo assay, with a range of 3.26–1121.96 S/CO (Fig. 3), indicating that this assay can also detect diverse HIV specimens. As expected, specimens that were tested in both assays had 100% concordant results.

In this study, new group N, subtype H, U/URF, and CRF01, 02, 06, 09, 11, 13, 18, 22, 37, and 43 sequences are reported that further expand the known genetic diversity of HIV-1. The RT and HIV Combo assays successfully detected these geographically and genetically diverse HIV-positive clinical specimens. Previous studies that challenged RT and HIV Combo assay performance with diverse strains of HIV relied on virus isolate cultures for some rare strains, such as groups N and P.^4,5,15^ In contrast to native specimens, virus culture isolates do not contain HIV-1 antibodies and therefore do not completely represent the complexity of human specimens. The results of this study indicate that both assays can detect diverse strains of HIV-1, accommodating both sequence and geographical diversity. Both assays were developed before groups N and P strains were identified and can readily detect these new strains without any further modification to the assays, which suggests that these assays will continue to be able to detect newly emerging strains of HIV as they arise. As HIV-1 diversity continues to present new challenges for diagnostic assays, viral surveillance remains essential for identification of specimens from divergent strains of HIV-1 to ensure diagnostic tests continue to accurately detect infections regardless of where they are acquired.

## Sequence Data

The polymerase sequences have been deposited in Genbank under accession numbers MG012032-MG012216 and envelope sequences have been deposited under accession numbers MG022442-MG022621.
